# Transcriptome profiling reveals the roles of pigment formation mechanisms in yellow *Paeonia delavayi* flowers

**DOI:** 10.1007/s00438-022-01973-4

**Published:** 2022-12-29

**Authors:** Hongzhu Zou, Lin Zhou, Lulu Han, JiHang Lv, YingHua Jia, Yan Wang

**Affiliations:** Key Laboratory of Tree Breeding and Cultivation, State Forestry Administration, Research Institute of Forestry, Chinese Academy of Forestry, Beijing, 10091 China

**Keywords:** Transcriptome, Carotenoid metabolic pathway, Flavonoid metabolic pathway, Flower colour, *Paeonia delavayi* var. *Lutea*

## Abstract

The yellow colour of ornamental varieties of tree peony originated from *Paeonia delavayi*. However, but *P. delavayi* and *Paeonia suffruticosa* belong to different subgroups, so hybridization is difficult and results in a long breeding cycle. However, no comprehensive transcriptomic profiling has focused on the colour formation mechanisms of yellow tree peony petals. Analysing the colour formation mechanism of yellow petals in *P. delavayi* is very important for directional molecular breeding. In this study, the transcriptional map of yellow pigment development in petals was used to analyse the mechanism of petal colour formation. We analysed the genes related to the metabolism of flavonoids and carotenoids and the transcription factors (TFs) involved in *P. delavayi* var. *lutea* (pure yellow individual) yellow pigment development using transcriptome sequence profiling. Transcriptome sequence profiles revealed three and four differentially expressed transcripts (DETs) involved in flavonoid biosynthesis and carotenoid biosynthesis, respectively. An analysis of DETs in the flavonoid pathway showed that chalcone synthase (*CHS*) and chalcone 2´-glucosyltransferases (THC*2’GT*) act in synergy to synthesize isosalipurposide (ISP). *CHS* and flavonol synthase (*FLS*) synergistically synthesize quercetin and kaempferol. DEG analysis of the carotenoid pathway revealed that phytoene synthase (*PSY*), carotenoid isomerase (*CRTISO*) and β-carotene hydroxylases (*CHYB*) play a key role in regulating lutein formation, and carotenoid cleavage dioxygenase (*CCD*) plays an important role in the degradation of carotenoids. These two pathways may be regulated by TF families such as *bHLH*, *ARF*, and *MYB*. The results of the transient overexpression of genes showed that *CHS* and *CHI* are regulated by *PdMYB2*. In this study, the molecular mechanism of ISP synthesis was analysed in depth, and the complete metabolic pathway of carotenoids in *Paeonia* L. was reported for the first time. By studying the formation mechanism of yellow pigment in *P. delavayi* petals, a breeding strategy for improving flavonol and carotenoid contents and reducing anthocyanin synthesis by genetic engineering was suggested.

**Supplementary Information:**

The online version contains supplementary material available at 10.1007/s00438-022-01973-4.

## Introduction

Tree peony (*Paeonia suffruticosa* Andrew) is a traditional Chinese flower that is loved by people all over the world because of its beautiful flowers. Tree peonies with yellow flowers have always been a valuable variety and are also a current breeding direction. *P. delavayi* is a special species due to its various petal colours, including yellow, orange, red, purple–red and dark red (Li et al. 2011). *P. delavayi* var. *lutea* is its yellow variation. The more successful yellow tree peony varieties on the market, such as the ‘High Noon’, ‘Alice Harding’ and Itoh hybrid series, have a *P. delavayi* var. *lutea* pedigree. Stable pure yellow petals have made *P. delavayi* var. *lutea* a suitable material for researching the formation of yellow flowers in tree peony. *P. delavayi* and *P. suffruticosa* belong to different subgroups, which makes hybridization difficult and results in a long breeding cycle. Hybrids also easily inherit the characteristics of small flowers and drooping flower heads from *P. delavayi*. Therefore, molecular breeding is the best way to obtain yellow-flowered tree peony. However, the molecular mechanism of yellow colour synthesis in *P. delavayi* petals is not clear. Analysing the molecular regulatory mechanism of yellow colour synthesis in *P. delavayi* petals is helpful for identifying the key genes involved in yellow anthocyanin synthesis and laying a foundation for the molecular breeding of tree peony.

Carotenoids and flavonoids are two main categories of yellow pigments in nature. In carotenoids, the main yellow substances are cryptoxanthin, zeaxanthin, lutein, violaxanthin and neoxanthin (Fraser and Bramley 2004). In flavonoids, flavones and flavonols are milky white to light yellow, while chalcones and aurones are pure yellow to dark yellow (Tanaka et al. 2010). In tree peony, the glycosides kaempferol, luteolin, apigenin and isosalipurposide (ISP) were the main flavonoids investigated. ISP is a kind of chalcone that is the main pigment component of *P. delavayi* var. *lutea* petals (Li et al. 2009a; Zhou et al. 2011). A few genes have been identified to be involved in the formation of ISP, including chalcone synthase (*CHS*) and chalcone2´-glucosyltransferases (THC*2’GT*). In ISP biosynthesis, *CHS* catalyses 1-molecule coumaroyl-CoA and 3-molecule malonyl-CoA to form 2´,4´,6´,4-tetrahyroxychalcone (THC), and THC generates a stable ISP in the vacuole under the action of THC*2’GT* (Yoshio et al. 2002). THC produces naringenin (colourless) under the action of chalcone isomerase (*CHI*), which is the precursor of anthocyanin, copigment flavone and flavonol biosynthesis. Naringin is catalysed by flavanone 3-hydroxylase (*F3H*) to synthesize dihydrokaempferol, followed by the synthesis of kaempferol (light yellow) and quercetin (light yellow) by flavonol synthase (*FLS*) (Bogs et al. 2005; Schijlen et al*.* 2006; Sharma and Dixon 2005). In addition, 14 carotenoids were identified by Zou et al. (2021) in *P. delavayi* var. *lutea*, and the yellow pigment with the highest content was lutein. In lutein biosynthesis, geranylgeranyl diphosphate (GGPP) first generates 15-cis-phytoene under the action of phytoene synthase (*PSY*), and then lycopene is generated under the successive action of phytoene desaturase (*PDS*), ζ-carotene desaturase (*ZDS*) and carotenoid isomerase (*CRTISO*). Under the catalysis of lycopene ε-cyclase (*LCYE*) and lycopene β-cyclase (*LCYB*), lycopene produces α-carotene, and then β-carotene hydroxylases (*CHYB*) catalyse the production of zeinoxanthin and zeioxanthin, which are catalysed by ε-carotene hydroxylases (*CHYE*) to form lutein. On the other hand, the degradation of carotenoids is accomplished by carotenoid cleavage dioxygenase (*CCD*) to decompose carotenoids and remove auxiliary carotenoids (Hannoufa and Hossain 2012; Li and Yuan 2013).

Because ISP is only found in a few species, such as carnation (Yoshida et al. 2004), cyclamen, catharanthus (Harborne 1966), kangaroo paw (Brkljaca et al. 2015) and peony (Li et al. 2009a, b; Zhou et al. 2011), *THC2’GT* has rarely been studied. THC is the substrate for almost all flavonoids (Ogata et al. 2004), and we do not know who is competing with THC*2’GT* in *P. delavayi* var. *lutea*. It is difficult to understand the formation of the ISP or the other underlying mechanism of floral development in tree peony. In the past, there were no reports of carotenoid-related genes in tree peony, and the carotenoid metabolic mechanism in tree peony is not clear. In this study, considering the four developmental stages of *P. delavayi* var. *lutea* petals, RNA-seq and full-length ISO-seq were used to analyse the transcriptome sequences and the gradual change in petal colour of *P. delavayi* var. *lutea*. Critical changes were found in the expression of genes involved in carotenoid and flavonoid pigment biosynthesis. We identified structural genes related to carotenoid and flavonoid biosynthesis during the flower development process and further explored key structural genes and transcription factor (TF) interactions in these pathways. The findings provide a systems-level context for further studies on the multigene regulation of carotenoid and flavonoid biosynthesis in *P. delavayi*.

## Materials and methods

### Plant materials and RNA preparation

**Fig. 1:**
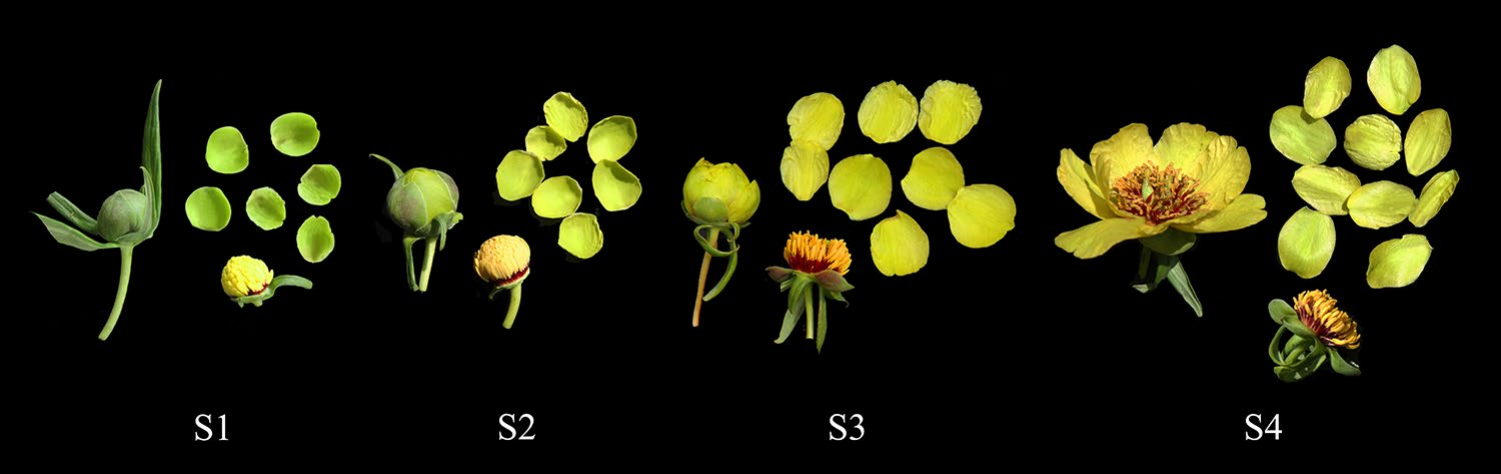
Four blooming periods of *P. delavayi* var. *lutea* flowers. S1: Unpigmented tight bud, the petals are completely green. S2: Slightly pigmented soft bud, the petals began to change from green to yellow. S3: Initially opened flower, the stamens are not visible, and the petals have completely turned yellow. S4: Fully opened flower, the stamens are completely exposed, and the petals are a lighter yellow than those in S3

*P. delavayi* var. *lutea* is an important breeding resource for tree peonies with yellow flowers. All the materials were collected from flowers of tree peony in Shangri-La County, Yunnan, China (E: 99°34′60″; N: 27°58′7″). The development of yellow flowers was distinguished into four stages from buds to open flowers: unpigmented tight bud (S1), slightly pigmented soft bud (S2), initially opened flower (S3), and fully opened flower (S4) (Fig. 1). Only petals were collected as experimental materials; they were immediately frozen in liquid nitrogen and then stored at − 80 °C.

Total RNA was extracted from the petals of the four developmental stages mentioned above using an EASYspin Plus Plant RNA Kit (No. RN38; Aidlab Biotechnologies Co., Ltd, Beijing, China) according to the manufacturer’s protocols. The RNA integrity was determined by an Agilent 2100 bioanalyzer and agarose gel electrophoresis. The purity and concentration of the RNA were determined by a Nanodrop microspectrophotometer (Thermo Fisher). Samples with intact electrophoresis bands, an RNA integrity number (RIN) > 7.0 and an RNA concentration > 50 ng/µL were used for transcriptome sequencing and real-time fluorescence quantitative PCR verification.

### Illumina and PacBio sequencing, functional annotation and classification, and identification of differentially expressed transcripts (DETs)

cDNA library construction and sequencing were performed by Gene Denovo Biotechnology Co. (Guangzhou, China). The Illumina cDNA libraries were sequenced on an Illumina Nova-seq 6000. The PacBio cDNA libraries were sequenced on the PacBio Sequel II platform using P6-C4 chemistry with 10 h movies. The raw sequencing reads of cDNA libraries were classified and clustered into consensus transcripts using the SMRT Link v5.0.1 pipeline (Gordon et al. 2015) supported by Pacific Biosciences. The raw reads were filtered to obtain high-quality reads. The filtering rules included removing reads containing adapters, removing reads containing more than 10% unknown nucleotides (N), and removing low-quality reads containing more than 40% low-quality (*Q*-value ≤ 20) bases. The high-quality clean reads were mapped to ribosomal RNA (rRNA) to identify residual rRNA reads. The high-quality clean reads without rRNA were mapped to the reference transcriptome using the short read alignment tool Bowtie2 (Li et al. 2009b) with default parameters, and the mapping ratio was calculated. The calculation formula is Mapping Ratio = (Number of Uniquely Mapped Reads + Number of Multiple Mapped Reads)/Total Number of Reads. The gene abundances were calculated and normalized to RPKM (reads per kb per million reads) (Mortazavi et al. 2008). The calculation formula is *RPKM* = 10^6^C/(*NL*/10^3^).

The basic annotation of isoforms included protein functional annotation, pathway annotation, COG/KOG functional annotation and Gene Ontology (GO) annotation. For annotation, isoforms were BLAST analysed against the NCBI nonredundant protein (Nr) database (http://www.ncbi.nlm.nih.gov), the Swiss-Prot protein database (http://www.expasy.ch/sprot), the Kyoto Encyclopedia of Genes and Genomes (KEGG) database (http://www.genome.jp/kegg), and the COG/KOG database (http://www.ncbi.nlm.nih.gov/COG) with the BLASTx program (http://www.ncbi.nlm.nih.gov/BLAST/) at an *E*-value threshold of 1e−5 to evaluate sequence similarity with genes of other species. Gene Ontology (GO) annotation was performed with Blast2GO software (Conesa et al. 2005) compared to the Nr annotation results of the isoforms. The open reading frames (ORFs) were detected using ANGEL (Shimizu et al. 2006) software for the isoform sequences to obtain the coding sequences (CDS), protein sequences, and UTR sequences. Protein domain prediction was performed by aligning the protein sequences of the isoforms to the Pfam database (version 26.0) with the Pfam_Scan program (Finn et al. 2016) and SMART database (version 06/08/2012) with the HMMER-profile hidden Markov models for biosequence analysis (http://hmmer.org/) program to obtain protein domain annotations. For plants, the protein coding sequences of the isoforms were aligned by hmmscan to Plant TFdb (http://planttfdb.cbi.pku.edu.cn/) to predict TF families. To identify DETs across samples or groups, the edgeR package (https://bioconductor.org/packages/release/bioc/html/edgeR.html) was used. The raw count data were imported into Bioconductor package edgeR (Robinson et al. 2010) in the R language to identify the differentially expressed genes and to calculate the RPKM of each gene. When calculating the differentially expressed genes, a gene was retained only if it was expressed at a count-per-million (CPM) above 0.5 in at least two samples. We identified genes with a fold change ≥ 2 and a false discovery rate (FDR) < 0.05 in a comparison as significant DETs. DETs were then subjected to enrichment analysis of GO functions and KEGG pathways.

### Real-time fluorescence quantitative PCR verification of DETs in *P. delavayi* var. *lutea*

Fourteen DETs (five genes in flavonoid synthesis, five genes in carotenoid synthesis, two genes in the *MYB* family, and two genes in the *bHLH* family) (Supplementary Table 1) involved in petal development of *P. delavayi* var. *lutea* were selected, and primers were designed using Primer3web (https://primer3.ut.ee/) online primer design software. The basic principles of design are as follows: primer length 17–25 bp, GC content 45–55%, TM value 60–68 ℃, and product length 80–1150 bp. The quantitative fluorescence PCR determination instrument was an Applied Biosystems StepOnePlus Real-Time PCR System, and the reagent was TaKaRa TB Green® *Premix* Ex Taq™ (Tli RnaseH Plus). The reaction system was as follows: TB Green *Premix* Ex Taq (Tli RnaseH Plus) (2 ×), 10 μl; forward and reverse primers (10 μM), 0.4 μl, for a total of 0.8 μl; correction solution ROX Reference Dye (50 ×), 0.4 μl (< 100 ng); and sterilized water, 6.8 μl, for a total volume of 20 μl. The 2^−△△^Ct method was used to calculate the expression, as described by Livak and Schmittgen (2002), and the internal reference was *PsPP2A*. Three biological replicates and two technical replicates were set for each quantitative test. All data are expressed as the mean ± SD after normalization.

### Functional validation of *MYB* transcription factors

To validate the MYB TFs, transient overexpression assays were carried out in the leaves of *Nicotiana benthamiana* according to the methods proposed by Lu et al. (2019). Briefly, cDNAs were synthesized from the total RNA of petals of *P. delavayi* var. *lutea* in the S2 stage. The target genes were cloned into the pCAMBIA1302 vector (Supplementary Table 1). The vector was under the control of a 35S promoter. Subsequently, the constructs harbouring the cloned target gene were transformed into *Agrobacterium tumefaciens* strain GV3101 by heat shock. Then, the Agrobacterium cell cultures were infiltrated into the leaves of *N. benthamiana.* The blank vector was injected into different leaves of the same tobacco as a control, and repeated experiments were carried out in three tobacco plants. After 60 h, the infiltrated leaves were harvested, and the expression of *NtCHS* and *NtCHI* was analysed by qRT–PCR (Supplementary Table 1).

## Results

### Transcriptomic changes in *P. delavayi* var. *lutea* during yellow pigment development

A total of 32.69 Gb of raw sequence data were generated by 28,134,899 subreads based on RNA-seq using PacBio ISO-seq. After being classified and clustered into the consensus transcript, CCSs (circular consensus sequences) containing 581,257 reads were obtained, which included 60.51% FLNC (full-length nonchimeric) and 39.49% nFL (non-full-length) sequences. Consequently, a total of 351,703 high-quality FLNC reads were obtained, and their average length was 2352 bp. The high-quality sequences were made nonredundant, and a full-length transcript of 103,240 bp was obtained. The total length of the isoform sequence was 246,339,799 bp, the length of the longest isoform was 14,187 bp, the length of the shortest isoform was 57 bp, the average length was 2386.09 bp, the N50 length was 3,373 bp, and the GC content was 39.82% (Supplementary Table 2).

The raw 154.75 Gb of data from 12 samples were sequenced using the Illumina Nova-Seq 6000 platform. The raw data range of a single sample was 11.26–14.16 Gb, and a total of 1,031,643,140 raw reads were obtained. The raw read number of a single sample ranged from 75,039,494 to 99,674,848. After removing impurities and ribosomal sequences, 1,017,213,846 clean reads in total and 75,039,494–97,552,438 clean reads per single sample were obtained. The lowest percentage of clean reads was 97.87%. The total number of unique mapped reads was 103,341,297, the range of unique mapped reads in a single sample was 6,793,163–10,741,602, the total number of multiple mapped reads was 849,525,052, the number of multiple mapped reads in a single sample ranged from 60,261,006 to 84,302,306, and the lowest comparison rate was 91.63% (Supplementary Table 3). All raw high-throughput sequence data have been deposited in the NCBI Sequence Read Archive (SRA) database under accession number PRJNA772706.

### Expression profile of the transcriptome of yellow petals in relation to flower development

**Fig. 2:**
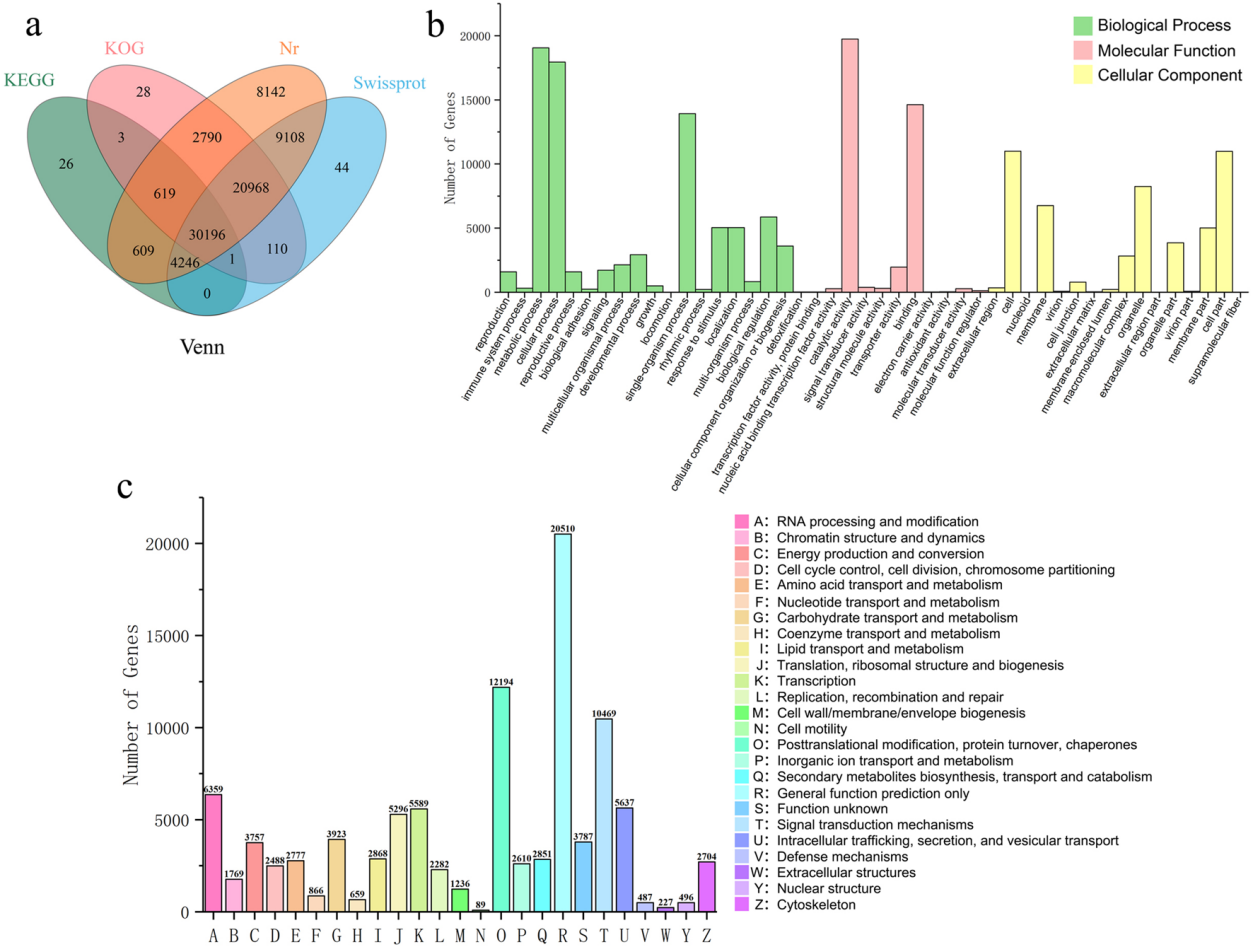
Functional annotation and classification of isoforms in *P. delavayi* var. *lutea*. a Venn diagram of annotations across four databases. **b** Gene Ontology classification analysis of *P. delavayi* var. *lutea*. **c** KOG classification of isoforms from *P. delavayi* var. *lutea*

Four public databases, Nr, Swissport, KOG and KEGG, were used to annotate all isoforms based on sequence similarity. There were 103,240 isoforms and 76,889 isoform annotations in the full-length transcript. Among them, 76,677 isoforms were annotated to the Nr database, 64,672 isoforms were annotated to the Swissport database, 35,699 isoforms were annotated to the KOG database, and 35,699 isoforms were annotated to the KEGG database. There were 26,351 isoforms (25.52% of the full-length transcripts) that did not significantly match any of the sequences in the public databases. These new genes with unknown functions are worthy of further study. The 4 database annotations and their overlaps were visualized in the form of a Venn diagram (Fig. 2a), with 30,196 common items in the 4 databases.

According to the Nr annotation information, the GO functional annotations were obtained, and the gene annotations of isoforms were standardized (Fig. 2b). A total of 31,669 isoforms were divided into 3 categories: molecular function, cellular component and biological process. In the molecular function category, binding, catalytic activity and transporter activity accounted for a relatively high proportion of the terms. In the cellular component category, cell, cell part, organelle, organelle part, membrane, membrane part and macromolecular complex accounted for the highest proportion of the terms. In the biological process category, cellular process, metabolic process, single-organism process, biological regulation, cellular component organization or biogenesis, developmental process, localization and response to stimulus accounted for a relatively high proportion of the terms.

The assembled isoforms were annotated with Cluster of Orthologous Groups of proteins (KOG). A total of 101,927 isoforms were assigned to 24 KOG clusters, and the largest category was general function prediction only, followed by posttranslational modification, protein turnover, chaperones, signal transduction mechanisms, RNA processing and modification, transcription, translation, ribosomal structure and biogenesis (Fig. 2c).

### Identification of DETs

**Fig. 3:**
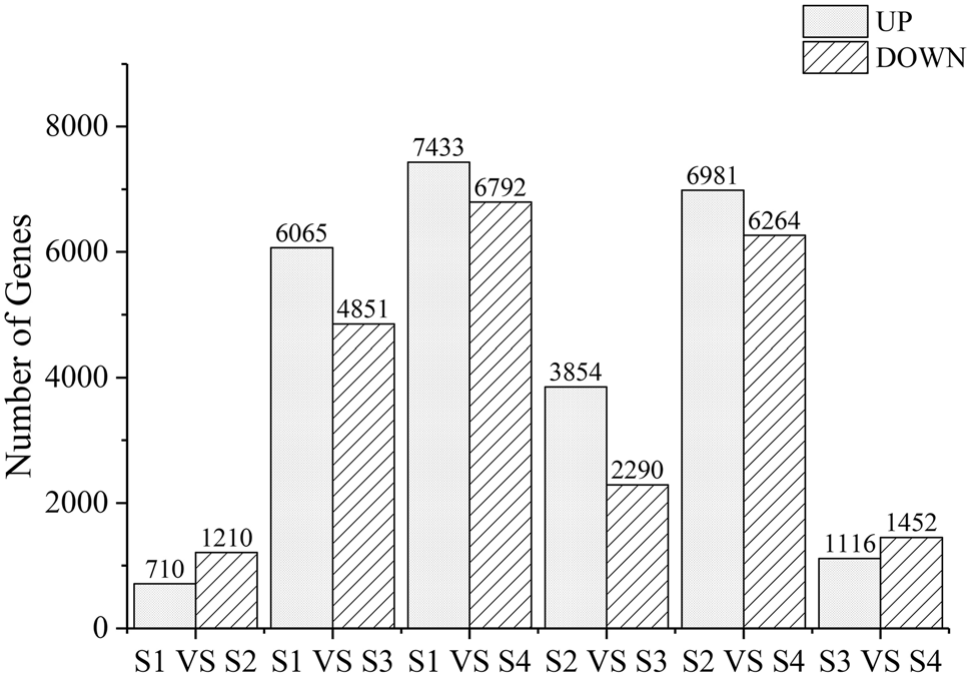
DETs involved in the four different flower developmental stages of *P. delavayi* var. *lutea*

To identify the DETs during flower development, we compared the transcription levels of each isoform at different developmental stages, and a total of 22,912 genes were differentially expressed at different stages. In the comparison between S1 and S2, a small number of DETs were detected, with 710 upregulated transcripts and 1210 downregulated transcripts. In the comparison between S1 and S3, 6065 upregulated transcripts and 4851 downregulated transcripts were detected. The number of DETs detected between S1 and S4 was the largest, with 7433 upregulated transcripts and 6792 downregulated transcripts. In the comparison between S2 and S3, 3854 upregulated transcripts and 2290 downregulated transcripts were detected. A large number of transcripts were also detected in the comparison between S2 and S4, including 6981 upregulated transcripts and 6264 downregulated transcripts. In the comparison between S3 and S4, the number of DETs detected was lower, with 1116 upregulated transcripts and 1452 downregulated transcripts (Fig. 3).

TFs play important roles in regulating colour change and pigment synthesis. By analysing the transcriptome data, a total of 55 TF families were predicted by hmmscan alignment between the predicted protein sequence and plantTFdb. The bHLH and ARF families were the most abundant TF families during the *P. delavayi var. lutea* yellow pigment development process, followed by the MYB_related, C3H, GRAS, FAR1, C2H2 and MYB families. The bHLH, ARF, C3H, and GRAS families had more upregulated genes during the whole flower blooming process. The MYB_related and MYB families had more downregulated genes during the S1–S3 stage of the yellow pigment development process, while S3–S4 had more upregulated genes after the flowers had turned yellow (Table 1).

**Table 1:** Differentially expressed transcription factors (TFs) in the S1-S4 stage

TF family	Total	S1 vs. S2		S2 vs. S3		S3 vs. S4		Description
		Up	Down	Up	Down	Up	Down	
*bHLH*	191	13	16	18	11	26	3	Helix-loop-helix DNA-binding domain
*ARF*	161	11	2	12	11	5	10	Auxin response transcription factors
*MYB_*related	160	2	18	10	12	5	1	*MYB*-related protein
*C3H*	132	7	3	3	4	3	4	Zinc finger CCCH domain-containing protein
*GRAS*	117	12	2	6	8	3	4	GAI, RGA, and SCR
*FAR1*	115	2	3	5	6	3	3	Far-red impaired response 1
*C2H2*	113	3	1	1	6	7	3	Zinc finger C2H2 domain-containing protein
*MYB*	93	7	8	7	12	8	4	MYB-related protein
*HD-ZIP*	93	7	2	8	5	8	3	Homeobox leucine zipper
*bZIP*	92	1	4	3	4	5	1	Basic region leucine zipper
*TALE*	77	2	12	1	8	1	2	Three-amino acid loop extension
*Trihelix*	70	2	1	2	3	6	1	Three Helix-loop-helix DNA—binding domains
*ERF*	66	1	6	9	7	9	1	Ethylene-responsive transcription factor
*WRKY*	63	2	3	0	2	1	1	*WRKY* DNA-binding domain
*SBP*	59	21	0	14	3	7	2	Squamosa promoter Binding Protein
*NAC*	59	6	1	3	5	2	3	NAC domain-containing protein
*TCP*	49	4	9	1	12	2	2	Teosinte branched 1, cycloidea, and PCF1
*GATA*	47	3	4	3	3	2	2	GATA domain-containing protein
other TFs	510	24	19	16	26	19	12	
total	2267	130	114	122	148	122	62	

The analysis of transcription factors is based on isoforms

### Analysis of flavonoid pathway gene expression during flower development

**Fig. 4:**
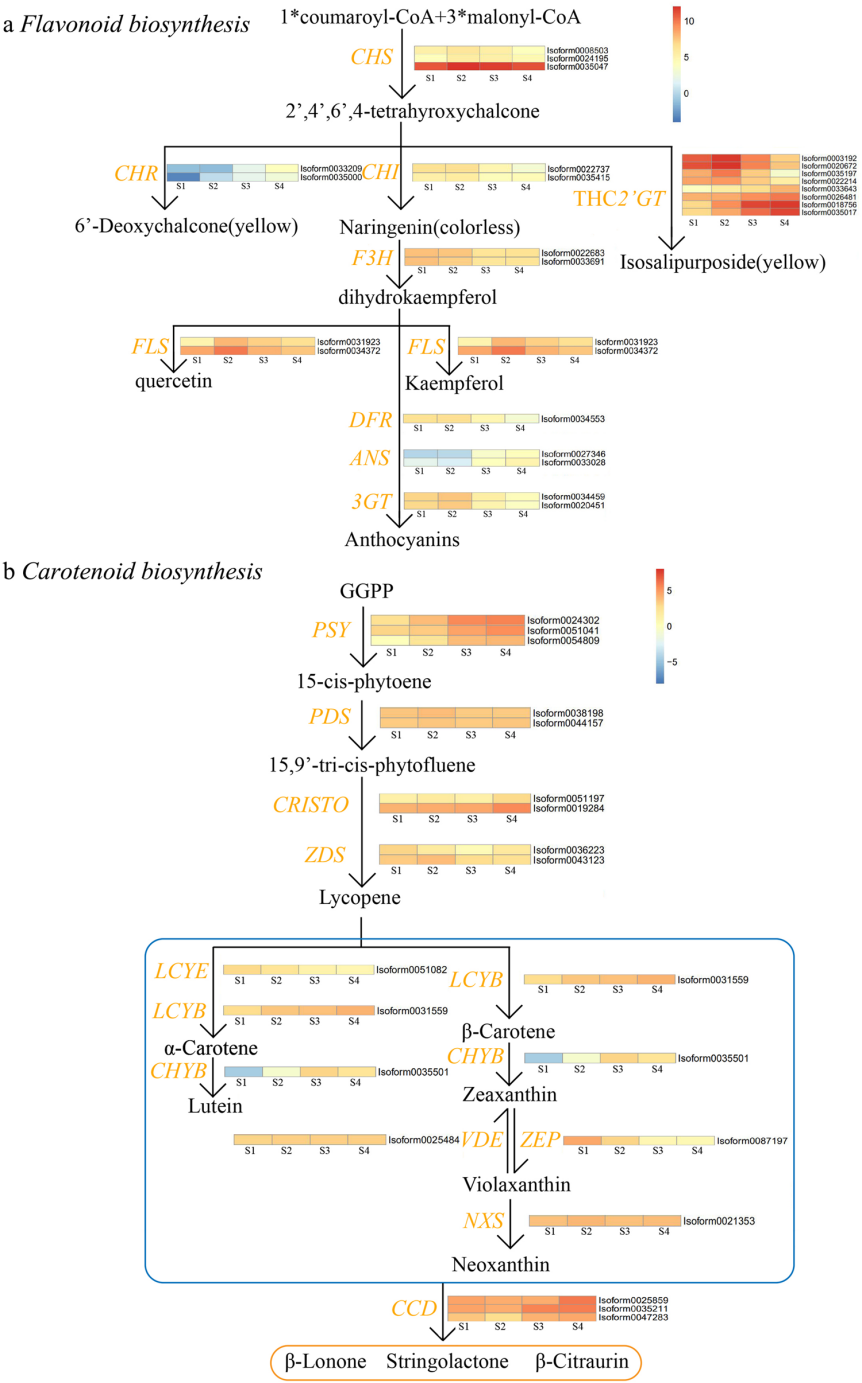
Expression heatmap of DETs for pigment metabolism in *P. delavayi* var. *lutea* during yellow pigment development. The mean FPKM values for the structural s were calculated from three biological replicates for each sampling point (S1, S2, S3, and S4). The progression of the colour scale from blue to red represents an increase in the FPKM values. **a** DETs involved in flavonoid biosynthesis. *CHS* chalcone synthase, *CHR* chalcone reductase, THC*2’GT* chalcone2´-glucosyltransferases, *CHI* chalcone isomerase, *F3H* flavanone 3-hydroxylase, *FLS* flavonol synthase, *DFR* dihydroflavonol 4-reductase, *ANS* anthocyanidin synthase, and *3GT* 3-glucosyltransferase. **b** DETs involved in carotenoid biosynthesis. GGPP geranylgeranyl diphosphate, *PSY* phytoene synthase, *PDS* phytoene desaturase, *CRTISO* carotenoid isomerase, *ZDS* ζ-carotene desaturase, *LCYB* lycopeneβ-cyclase, *LCYE* lycopene ε-cyclase, *CHYB* β-carotene hydroxylases, *ZEP* zeaxanthin epoxidase, *VDE* violaxanthin de-epoxidase, *NXS* neoxanthin synthase, and *CCD* carotenoid cleavage dioxygenase

To identify the key genes involved in flavonoid biosynthesis, all isoforms in the flavonoid biosynthesis pathway were searched in the KEGG database, and a metabolic map was compiled that showed multiple transcripts of enzymes involved in the flavonoid biosynthesis pathway. Zhou et al. (2011) determined the composition of yellow pigment in *P. delavayi* and considered that the main flavonoid pigments were chalcone, flavones and flavonoids, including ISP, as well as kaempferol, quercetin, isorhamnetin, chrysoeriol and apigenin, of which the most important chromogenic substance was ISP. As shown in the figure, the biosynthesis of quercetin and kaempferol involved *CHS* (seven isoforms), *CHI* (four isoforms), *F3H* (five isoforms) and *FLS* (eight isoforms). In addition, the key enzymes chalcone reductase (*CHR*, three isoforms), *DFR* (two isoforms), *ANS* (two isoforms) and 3-glucosyltransferase (*3GT*, six isoforms) in the flavonoid metabolic pathway were identified. *THC2'GT* is essential for the biosynthetic pathway of ISP, which converts THC to ISP and stabilizes it in vesicles. However, this pathway was only found in a few species, such as *D. caryophyllus* and *Paeonia potaninii* var. *Trollioides* and *Catharanthus* (Harborne 1966; Yoshida et al. 2004). Thus, *THC2'GT* is seldom studied in plants and has not been accurately annotated in the KEGG database. In this paper, in the transcriptome data, a total of 37 isoforms belonging to the GT family were predicted. Based on the expression data of the identified flavonoid pathway DETs in the transcriptome, a heatmap of the flavonoid metabolic pathway was constructed (Fig. 4a). *CHS*s, as the first structural gene in the flavonoid metabolic pathway, showed a high expression level from S1 to S4. The low expression of *CHR* indicated that 6-deoxychalcone is not the main yellow substance in *P. delavayi var. lutea*, which was consistent with the identification results of flower colour. *FLS* catalyses the formation of flavonols, and quercetin and kaempferol, important components of flower colour, need to be catalysed by *FLS*s, which is consistent with the high expression of *FLS*s. Some highly expressed genes were also identified among the candidate genes of THC*2'GT*s, which was much higher than that of the other two branches, suggesting that THC*2'GT*s played an important role in this pathway. Among these DETs, the expression of some genes increased significantly from S1 to S2, reached a peak in S2, and then decreased slowly in S3 and S4. The expression of another part of the gene increased continuously in S1–S4 and reached its peak in S4. This provided an important reference for the identification of the THC*2'GT* gene in the later stage. The high expression of THC*2'GT* made the ISP pathway compete strongly with the anthocyanin pathway. The expression levels of *DFR*s, *ANS*s and *3GT*s were all low, and a large amount of THC was converted into ISP instead of anthocyanin.

### Analysis of carotenoid pathway gene expression during flower development

To identify the key genes involved in carotenoid biosynthesis, all isoforms involved in the carotenoid biosynthesis pathway were searched in the KEGG database, and metabolic maps were compiled that showed multiple transcripts of enzymes involved in the carotenoid biosynthesis pathway. Upstream genes of the carotenoid biosynthesis pathway *PSY* (eight isoforms), *PDS* (two isoforms), *CRTISO* (six isoforms), and *ZDS* (four isoforms) were identified. Downstream structural genes of the carotenoid biosynthesis pathway *LCYE* (six isoforms), *LCYB* (three isoforms), *CHYB* (seven isoforms), *VDE* (3 isoforms), *ZEP* (three isoforms), *NXS* (one isoform), and *CCD* (seven isoforms) were identified. Based on the expression data of the identified carotenoid pathway DETs in the transcriptome, a heatmap of the carotenoid metabolic pathway was constructed (Fig. 4b). The results indicated that 12 isoforms encoding 6 enzymes showed marked changes in expression during the stages of flower development; among these isoforms, isoforms encoding *PSY*, *CRTISO*, *CHYB*, and *CCD* all showed upregulated expression during flower development from S1 to S4, while isoforms encoding *LCYE* and *ZEP* showed downregulated expression. Therefore, we speculated that the synthesis of lutein was more affected by *PSY, CHYB* and *CRTISO*. Lutein degradation was affected by *CCD*. The expression of all carotenoid pathway genes was extremely low, which indicated that carotenoids were not the main pigment component in the petals of *P. delavayi* var. *lutea*, which was consistent with the carotenoid content determination results (Zou et al. 2021).

### Validation of DEG expression in the transcriptome data

**Fig. 5:**
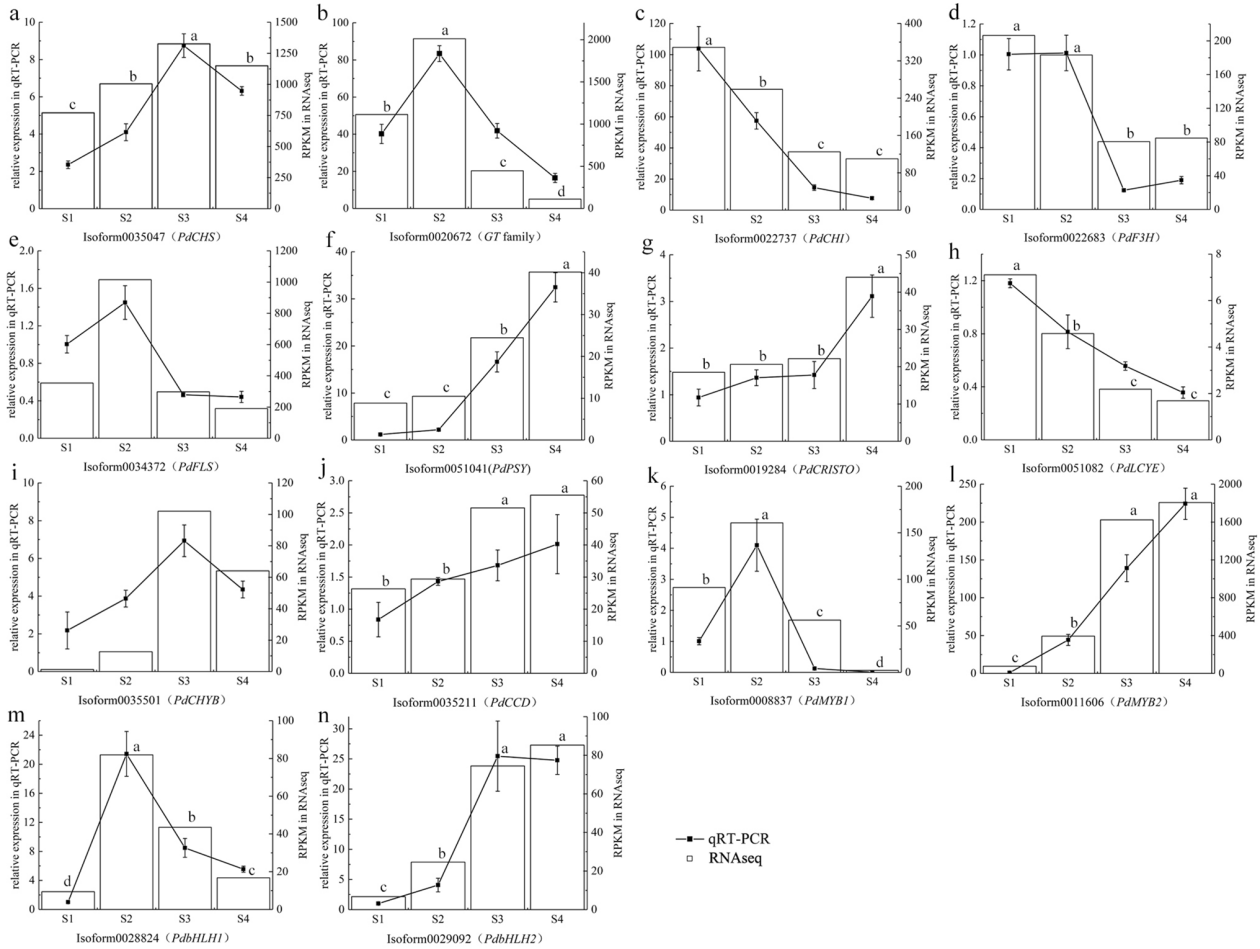
Comparison of RNA-seq and qRT–PCR analyses for 14 differentially expressed transcripts (DETs) during *P. delavayi* var. *lutea* yellow pigment development. **a**−**n** DETs associated with flavonoid biosynthesis (**a−e**), carotenoid biosynthesis (**f−j**), and TFs (**k−n**). The different letters indicate significant differences among the different sampling points (*P* < 0.05), and the error bars indicate the standard deviations

To validate the key DEG results (Fig. 5), the 14 DETs that showed the most significant differences in expression were selected: five flavonoid biosynthetic pathway genes (Fig. 5a–e), five carotenoid biosynthetic pathway genes (Fig. 5f–j), and four TFs (Fig. 5k–n). We analysed the evolution of gene expression during the yellow pigment development in *P. delavayi* var. *lutea* petals using qRT–PCR. The RNA-seq and qRT–PCR data were very closely correlated, and there was high consistency in the up- and downregulated expression of DETs.

### Functional validation of *PdMYB2*

**Fig. 6:**
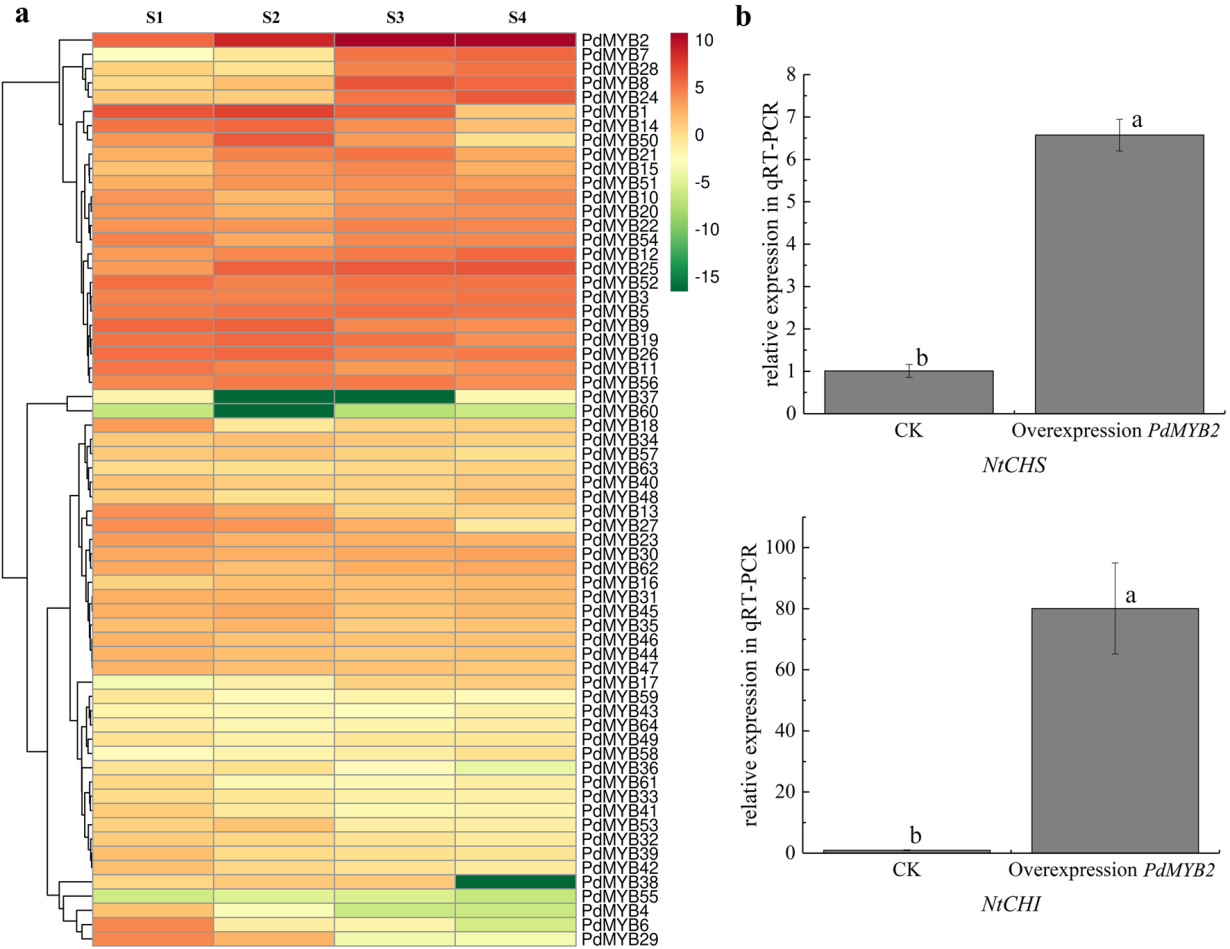
Functional Validation of *PdMYB2*. **a** Expression heatmap of *MYB* family members**.** The contents were log-transformed and used to generate a heatmap with the TBtools software package. **b** qRT–PCR analyses of *NtCHS* and *NtCHI* after overexpression of *PdMYB*. The different letters indicate significant differences among the different sampling points (*P* < 0.05), and the error bars indicate the standard deviations

The *MYB* TF family is one of the most influential TF families in the process of flower colour expression. We annotated all the MYB TFs and totally 64 MYB gene family members were obtained. We constructed an expression heatmap based on the transcriptome data. The results showed that the expression of *PdMYB2* (Isoform0011606) was significantly different in the 4 stages and was much higher than that of other MYB members (Fig. 6a). To validate the *MYB* TFs mentioned above, transient overexpression assays were carried out in *Nicotiana benthamiana* leaves. As expected, the transcript levels of the target genes *NtCHS* and *NtCHI* were significantly increased by *PdMYB2* overexpression in *N. benthamiana* leaves for 60 h. However, the colour of tobacco leaves did not change, which may be related to the lack of a substrate for THC in tobacco leaves, making it difficult to synthesize anthocyanins. The function of *PdMYB2* needs further verification. Furthermore, the expression of *NtCHI* was higher than that of *NtCHS*, which may be related to the cascade effect of genes (Fig. 6b).

## Discussion

Transcriptome analysis provides an important dataset for understanding the molecular genetic basis of *P. delavayi* var. *lutea* coloration. The RNA-seq and qRT–PCR results indicated that the expression of flavonoid biosynthesis genes (such as *CHS*, THC*2'GT* and *FLS*) leads to the production of flavonols, and the expression of carotenoid biosynthesis genes (such as *PSY*, *CRTISO* and *CHYB*) leads to the production of lutein. These two pathways may be regulated by TF families such as *bHLH*, *ARF*, and *MYB*. We preliminarily showed that *CHS* and *CHI* are regulated by *PdMYB2*. The synergy of carotenoids and flavonoid glycosides leads to golden yellow petals. This research suggests directions for the breeding of yellow tree peony. For breeders, future research should focus on improving the levels of ISP and lutein and reducing the synthesis of anthocyanins through genetic engineering.

### Transcriptome sequencing and annotation

Peonies with yellow flowers are rare and popular. Flower colour is regulated by gene expression. Understanding the molecular mechanism controlling yellow coloration during flower development will help to cultivate yellow peony flowers with high ornamental value. However, there is little genetic information available for *P. delavayi*. The popularity of RNA-seq and full-length ISO-seq provides great convenience for the study of plant transcripts, especially for transcriptome analysis without reference genomes (Wang et al. 2014; Loraine et al. 2013). The RNA-seq data can be used to analyse the differential gene expression during the synthesis of flavonoids and carotenoids in the petals of *P. delavayi var. lutea* and correct the full-length ISO-seq data. The full-length ISO-seq data can directly obtain the complete full-length transcripts from 5´ to 3´ and optimize the RNA-seq data to make the quantitative results more accurate. Combined, RNA-seq and full-length ISO-seq were used to comprehensively analyse the biosynthesis pathway of isoberellin and carotenoids in *P. delavayi* var. *lutea* and discover key genes, which laid the foundation for directional breeding of yellow flowers in tree peony.

In this work, a total of 103,240 isoforms from *P. delavayi var. lutea* were annotated by comparison with the public databases Nr, Swissport, KOG and KEGG. However, the remaining 26,351 isoforms did not significantly match any sequences in the public database. The function of these newly reported *P. delavayi* var. *lutea*-specific genes is unknown and will be the subject of future research.

### Carotenoid metabolic pathway genes were first reported in tree peony

To date, the genes of the carotenoid metabolism pathway in peony have not been reported. In the report by Zou et al. (2021), the main carotenoid in the S1 phase was lutein, and the metabolic flux in the S4 phase basically stops at (E/Z)-phytoene. From the expression heatmap data, the expression of *PSY* gradually increased, while the expression of downstream structural genes gradually decreased, which was consistent with the change in carotenoid content. *CCD* reduces the accumulation of carotenoids in plants by decomposing carotenoids into deauxiliary carotenoids (Li and Yuan 2013; Hannoufa and Hossain 2012). In this study, the expression of *CCD* was negatively correlated with the accumulation of lutein and was consistent with the flower colour phenotype. The results of this study show that the carotenoid synthesis pathway and its metabolism in *P. delavayi* var. *lutea* were basically complete, which means that there is another pigment pathway in tree peony that can be targeted gene breeding.

### Flavonoid accumulation in tree peony is regulated at the transcriptional level

ISP is the main source of yellow pigment in petals of *P. delavayi* (Zhou et al. 2011). *CHS* is an especially important enzyme in the flavonoid biosynthesis pathway. It catalyses the production of THC, the most important intermediate in the flavonoid biosynthesis pathway. *THC2'GT* is the most critical enzyme in the synthesis of ISP, and *THC2'GT* converts THC into chalcononaringein2´-*O*-glucoside, namely, ISP. In this study, Isoform0035047, annotated as *CHS*, continued to be highly expressed during the development of S1–S4, which also proved the important role of CHS in yellow petals. Itoh et al. (2002) found that the synthesis and accumulation of *THC2'GT* caused the yellow petals of *D. caryophyllus*. ISP has been detected in many plants and causes yellow flowers in *Corylopsis* spp. (Iwashina et al. 2009). Due to the lack of research on *THC2'GT* in plants, the transcriptome data were unable to directly annotate the gene. After selecting the genes annotated to the *GT* family and constructing the expression heatmap, it was found that Isoform0003192, Isoform0020672, Isoform0018756 and Isoform0035017 were highly expressed during flower development. The expression of Isoform0003192 and Isoform0020672 first increased and then decreased, while the expression of Isoform0018756 and Isoform0035017 consistently increased. Combined with the fact that flowers began to turn yellow at the S2 stage and were completely yellow at the S3 stage, this study suggested that Isoform0003192 and Isoform0020672 may be more critical genes in the process of ISP production. This study provides a basis for the final identification and verification of the *THC2'GT* gene. In *P. delavayi* var. *lutea* transcriptome data, the expression of genes annotated as *CHI* showed a decreasing trend, and the expression of downstream genes such as *DFR* and *ANS* was also extremely low. This decrease may be due to the competition between the ISP pathway and anthocyanin pathway, resulting in a decrease in the expression of *CHI*, so that the petals did not show red colour.

### Effects of MYB TFs in yellow *P. delavayi* flowers

Based on the above research on pigment and related metabolic pathway genes in *P. delavayi*, we inferred that the degradation and biosynthesis of pigments occurred throughout the entire process of the whole flowering in a dynamic form that was directly related to some TFs of key genes. MYB transcription factor family is the largest transcription factor family in plants, among which R2R3 MYB is the largest in plants (Cao et al. 2013). They are mainly involved in cell differentiation, hormone response, secondary metabolism, environmental stress and resistance to diseases and insect pests, and can regulate the biosynthesis of plant anthocyanins (Tang and Chen 2014). After transient expression of *PdMYB2* in tobacco leaves, the expression levels of *NtCHI* and *NtCHS* in tobacco were obviously up-regulated, but the color of tobacco leaves did not change. We speculated that the main pigment in tobacco leaves was chlorophyll, and the accumulation of flavonoid substrates and products was not enough to cause the color change. MYB genes regulating flavonoid synthesis pathway have been isolated from many plants, and their functions have been studied. Different MYB transcription factors can cause different kinds of pigment accumulation, and their regulated genes are different.

Gates et al. (2017) found a new R3-MYB transcription factor allele from *Iochroma* spp., which is closely related to flower color variation, and named *MYBL1*, which is a transcription inhibitor of MYB. The high expression of this gene is related to the down-regulation of multiple anthocyanin pigment pathway genes. Deng et al. (2021) found a R2R3-MYB transcription factor *MaMYB4* in banana (*Musas* PP.), which can combine with the promoters of CHS, ANS, DFR and bHLH, thus inhibiting their expression and reducing the accumulation of anthocyanins. The *MdMYB10* isolated from apple (*Malus* × *domestica*) by Espley et al. (2007) can induce anthocyanin accumulation in both homologous and heterologous systems, and co-express with two bHLH proteins, *MdbHLH3* and *MdbHLH33*. Because PdMYB2 is highly expressed in the petals of *P. delavayi*, but there is no visible red pigment in the petals of pure *P. delavayi*, we speculate that *PdMYB2* does not directly regulate the expression of *CHI*, but regulates the expression of downstream genes by regulating *CHS*. A R2R3-MYB gene *PsMYB12* was found in peony (Gu et al. 2019), studies have shown that *PsMYB12* and bHLH and WD40 proteins directly activate *PsCHS* expression to regulate the formation of petal spots. The continued verification of the function of *PdMYB2*, and whether *PdMYB2* interacts with bHLH, WD40 and directly interacts with CHS in *P. delavayi* are the key points of follow-up research.

### Breeding of yellow tree peony through genetic engineering

Based on the analysis of gene expression and pigment levels in the flavonoid metabolic pathway and carotenoid metabolic pathway of *P. delavayi* var. *lutea*, the mechanism behind yellow pigment production can be explained. First, the high expression of *CHS* and *THC2'GT* genes in the ISP pathway led to the accumulation of ISP in petals, and the expression of *FLS* led to the production of kaempferol and quercetin in petals. At the same time, the expression of *PSY*, *CRTISO*, *LCYE*, *LCYB* and *CHYB* in the carotenoid pathway regulates the production of lutein. The accumulation of these substances makes *P. delavayi* var. *lutea* appear bright yellow. Fukusaki et al. (2004) obtained white-flowered plants by silencing the *CHS* gene of blue *Torenia hybrida* by an RNA interference technique. Ohno et al. (2018) found that *DvCHS2* posttranscriptional gene silencing occurred in white petals rather than red petals in bicolour *Dahlia pinnata*. These results confirmed the important role of the *CHS* gene in anthocyanin accumulation. Yoshida et al. (2004) speculated that the difference in gene expression or enzyme activity of THC*2'GT* was the main reason for the difference in ISP content (ranging from 5.5 to 100%) in petals of different varieties of carnation and speculated that THC*2'GT* might not be regulated by a single gene but by multiple genes. Togami et al. (2011) cloned the cDNA of THC*2'GT* from *D. caryophyllus*, *C. persicum* and *Catharanthus roseus* and expressed it in *Petunia hybrida*, which caused ISP accumulation in the petals of *P. hybrida*. Giovanni et al*.* (2013) increased the content of lutein in tomato fruit by overexpressing *LCYB*. Yamamizo et al. (2010) determined that the high expression of *CHYB* was one of the main reasons for the accumulation of lutein and yellow coloration of petals of *P. hybrida*. In strawberry (*Fragaria* × *ananassa*), the upregulation of the expression of dioxygenase *FaCCD1* leads to a decrease in lutein accumulation (García-Limones et al. 2008). Therefore, we speculate that overexpression of *CHS* and THC*2’GT* and suppression of *CHI* will increase the accumulation of ISP. Overexpression of *LCYB* and *CHYB* and inhibition of *CCD* expression increased the accumulation of lutein. Gamsjaeger et al. (2011) used FT-Raman to analyse pigments on different coloured petals of pansy (*Viola* × *wittrockiana*), and the results showed that the flower colour of pansy was controlled by flavonoids (flavonoids and anthocyanins) and carotenoids. Cao et al. (2015) studied the metabolic process of carotenoids in citrus (*Citrus reticulata*), and the results showed that the changes in carotenoid accumulation had significant effects on redox modification, starch degradation and flavonoid/anthocyanin biosynthesis. These studies show that it is feasible to increase the content of ISP and lutein and reduce the synthesis of anthocyanins by genetic engineering. Whether the accumulation of carotenoids in *P. delavayi* var. *lutea* affects the content of flavonoids, and whether there is a cooperative relationship between them, is worthy of more in-depth study in the future.

## Supplementary Information

Below is the link to the electronic supplementary material.

Supplementary file1 (DOCX 17 KB)

Supplementary file2 (DOCX 14 KB)

Supplementary file3 (DOCX 16 KB)

## Data Availability

The data generated and analysed during the current study are available in the NCBI repository. https://www.ncbi.nlm.nih.gov/sra/PRJNA772706.
