# Supplementary material for: Transcriptome profiling reveals the roles of pigment formation mechanisms in yellow Paeonia delavayi flowers
Source: Mol Genet Genomics. 2022 Dec 29;298(2):375–87. doi: 10.1007/s00438-022-01973-4 (PMC9938063; doi:10.1007/s00438-022-01973-4)
Supplement: Supplementary file 1 — Supplementary file1 (DOCX 17 KB) [file 438_2022_1973_MOESM1_ESM.docx]

Supplementary Table.1 Primers used for real-time quantitative RT-PCR.

| Gene | Forword primer(5’ - 3’) | Reverse primer(5’ - 3’) | Annotation |
| --- | --- | --- | --- |
| Isoform20672 | AGCAGACATTTTGGCGTGGC | TGCGCTCCAGTCCATTTGCA | *GT* family |
| Isoform35047 | ACATTTCGCGGACCTTCGGA | TCAGCACCGACAATAACCGCA | *PdCHS* |
| Isoform22737 | AGGAGCTGGCCGAAGATGACAA | TCTCCAGTTGCACGCCATACT | *PdCHI* |
| Isoform0022683 | GCAAGAAAGGCGGGTTCATCGT | TCTGTTACTCGCTTCCACCCCT | *PdF3H* |
| Isoform0034372 | TTTGGCCAAGCAACCCACCA | TGGTGAGGGTGGACAAATCGGT | *PdFLS* |
| Isoform0051041 | ACACCGGCAGCTTTGGATAGGT | AGCCAACAGTTCCAGCGACA | *PdPSY* |
| Isoform0019284 | TGATGCCGAGGGAATACGCA | TCAAATGGCTCGGTTCCTGGTG | *PdCRISTO* |
| Isoform0051082 | TGGTGGTTATTGGCTGTGGTCCA | CCGCCAAACATGCTCAATACACCC | *PdLCYE* |
| Isoform0031559 | TCGACCTGGACTCTGCATGGAA | GCCGTACCACCAATCCCAACAA | *PdLCYB* |
| Isoform0035758 | TTGTGGCATGCTTCGCTGTG | TCCCAAACCAGCGCCAAAACA | *PdCHYB* |
| Isoform0035211 | GGTTGGCCGGAAACTTTGCT | ACCATGCCATCGCCATCAAACC | *PdCCD* |
| Isoform0008837 | AAGCCTCAACCTCGGACGTTCA | AGCCGTTCGTGTTTGGTGCA | *PdMYB1* |
| Isoform0011606 | TTCCCGGGACAGAGTTCAGA | ACGACTGAACAACGACGGGA | *PdMYB2* |
| Isoform0028824 | TCGGGAAAGCGCTAGTCCTTGA | ACCACCACCAACCTGCATGT | *PdbHLH1* |
| Isoform0029092 | TGAAGCTGCCGATCAAGCCT | AGAATACTCACCCGCGGCTTCT | *PdbHLH2* |
| *PsPP2A* | CGTGTTTGGATGTTCTCAAGGC | GGCGAGTGAGTTTTCAGTTGGA | Reference |
| Isoform0011606（clone） | ATGGACAAAAAACCATGCAGCTCC | TTAATCACCATTAAGTAAATGC | *PdMYB2* |
| Isoform0011606- pCAMBIA1302 | catggtagatctATGGACAAAAAACCATGCAGCTCC (NcoI) | agttcttctcctttactagtTTAATCACCATTAAGTAAATGC (SpeI) | PdMYB2 |
