# Supplementary material for: Transcriptome profiling reveals the roles of pigment formation mechanisms in yellow Paeonia delavayi flowers
Source: Mol Genet Genomics. 2022 Dec 29;298(2):375–87. doi: 10.1007/s00438-022-01973-4 (PMC9938063; doi:10.1007/s00438-022-01973-4)
Supplement: Supplementary file 2 — Supplementary file2 (DOCX 14 KB) [file 438_2022_1973_MOESM2_ESM.docx]

Supplementary Table.2 Statistics of Isoforms

| Total Number | Total length(bp) | Maximum Length(bp) | Minimum Length(bp) | Average Length(bp) | N50 Length(bp) | GC content |
| --- | --- | --- | --- | --- | --- | --- |
| 103240 | 246339799 | 14187 | 57 | 2386.09 | 3373 | 39.82% |
