# Supplementary material for: Transcriptome profiling reveals the roles of pigment formation mechanisms in yellow Paeonia delavayi flowers
Source: Mol Genet Genomics. 2022 Dec 29;298(2):375–87. doi: 10.1007/s00438-022-01973-4 (PMC9938063; doi:10.1007/s00438-022-01973-4)
Supplement: Supplementary file 3 — Supplementary file3 (DOCX 16 KB) [file 438_2022_1973_MOESM3_ESM.docx]

Supplementary Table.3 Statistics and filtrationsof the data from Illumina *RNA-Seq*

| sample | Raw data（Gb） | Raw Reads | Clean Reads | Clean reads rate(%) | Unique Mapped Reads | Multiple Mapped Reads | Mapping rate(%) |
| --- | --- | --- | --- | --- | --- | --- | --- |
| S1-1 | 11.26 | 75073002 | 74841402 | 99.69% | 9041493 | 60261006 | 92.60% |
| S1-2 | 14.16 | 94379678 | 93139078 | 98.69% | 10741602 | 75605591 | 92.71% |
| S1-3 | 12.62 | 84142072 | 83785938 | 99.58% | 10202000 | 67437407 | 92.66% |
| S2-1 | 13.45 | 89674178 | 87800146 | 97.91% | 8702380 | 73806983 | 93.97% |
| S2-2 | 11.44 | 76284496 | 75355234 | 98.78% | 7623894 | 63247437 | 94.05% |
| S2-3 | 12.63 | 84208682 | 83691136 | 99.39% | 9798922 | 68482493 | 93.54% |
| S3_1 | 14.95 | 99674848 | 97552438 | 97.87% | 8304171 | 84302306 | 94.93% |
| S3_2 | 13.38 | 89185588 | 88497182 | 99.23% | 8089274 | 75798143 | 94.79% |
| S3_3 | 13.47 | 89815244 | 87970950 | 97.95% | 8708447 | 71896377 | 91.63% |
| S4-1 | 11.26 | 75039494 | 74437934 | 99.20% | 6793163 | 63576496 | 94.53% |
| S4-2 | 12.09 | 80582568 | 78424914 | 97.32% | 6949308 | 66871838 | 94.13% |
| S4-3 | 14.04 | 93583290 | 91717494 | 98.01% | 8386643 | 78238975 | 94.45% |
